# Jeopardy of COVID-19: Rechecking the Perks of Phytotherapeutic Interventions

**DOI:** 10.3390/molecules26226783

**Published:** 2021-11-10

**Authors:** Priyanka Saha, Subhankar Bose, Amit Kumar Srivastava, Anis Ahmad Chaudhary, Rajiv Lall, Sahdeo Prasad

**Affiliations:** 1 Cancer Biology & Inflammatory Disorder Division, CSIR-Indian Institute of Chemical Biology, Kolkata 700032, WB, India; priaz12@rediffmail.com (P.S.); sbose33186@gmail.com (S.B.); amit@iicb.res.in (A.K.S.); 2 Department of Biology, College of Science, Imam Mohammad Ibn Saud Islamic University (IMSUI), Riyadh 11623, Saudi Arabia; aachaudhary@imamu.edu.sa; 3 Noble Pharma, LLC, 4602 Domain Drive, Menomonie, WI 54751, USA; lallr@vets-plus.com

**Keywords:** COVID-19, medicinal plants, inflammation, antioxidant, phytotherapy

## Abstract

The novel coronavirus disease (COVID-19), the reason for worldwide pandemic, has already masked around 220 countries globally. This disease is induced by Severe Acute Respiratory Syndrome-Coronavirus-2 (SARS-CoV-2). Arising environmental stress, increase in the oxidative stress level, weak immunity and lack of nutrition deteriorates the clinical status of the infected patients. Though several researches are at its peak for understanding and bringing forward effective therapeutics, yet there is no promising solution treating this disease directly. Medicinal plants and their active metabolites have always been promising in treating many clinical complications since time immemorial. Mother nature provides vivid chemical structures, which act multi-dimensionally all alone or synergistically in mitigating several diseases. Their unique antioxidant and anti-inflammatory activity with least side effects have made them more effective candidate for pharmacological studies. These medicinal plants inhibit attachment, encapsulation and replication of COVID-19 viruses by targeting various signaling molecules such as angiotensin converting enzyme-2, transmembrane serine protease 2, spike glycoprotein, main protease etc. This property is re-examined and its potency is now used to improve the existing global health crisis. This review is an attempt to focus various antiviral activities of various noteworthy medicinal plants. Moreover, its implications as prophylactic or preventive in various secondary complications including neurological, cardiovascular, acute kidney disease, liver disease are also pinpointed in the present review. This knowledge will help emphasis on the therapeutic developments for this novel coronavirus where it can be used as alone or in combination with the repositioned drugs to combat COVID-19.

## 1. Introduction

Coronavirus disease-19 (COVID-19, also known as SARS-CoV-2) has just become the hot cake internationally. This disease turned out as a pandemic in 2019 and has affected nearly 60 million people worldwide by 2021. This medical health emergency though initially starts with a mild symptomatic condition but in later stages creates an oxygen deficient environment into the human system, inducing a severe respiratory distress [1]. Several groups of researchers are working in collaboration to understand an in-depth physiological condition of the epidemiology of this disease. Not only the virologist, but also different group of scientists has directed their studies to COVID-19 research in this situation of pandemic to understand clinical complications among suffering patients and the role of upcoming secondary diseases associated with it. For instances, viral invasion causes severe cardiac injury in many patients attacking cardiomyocytes [2]. Additionally, severe rheumatological illness has also been reported post infection. Vasculitis and renal dysfunction are other related complications which are reported to be common complications [3]. Acute kidney infection and associated renal diseases are other reported complications post viral infections [4]. Most importantly, further research studies are warranted to better understand the impact of various environmental, physiological, and endogenous factors in the increase of mortality rates among the infected population.

**Figure 1:**
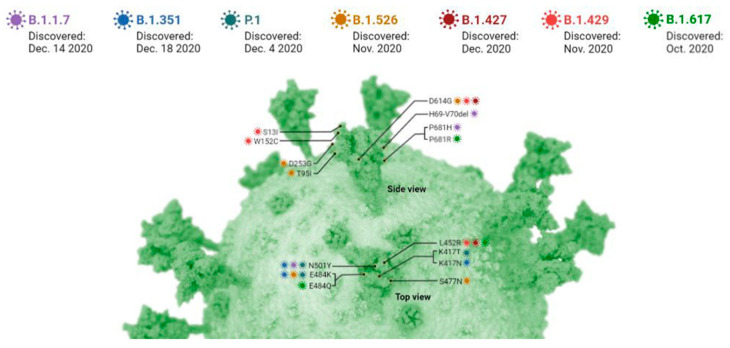
A snapshot of the rising variation in the strains of the SARS-CoV-2 due to mutations.

Upon studying various parameters, it was interestingly seen that oxidative stress plays a critical role in majority of patients. Oxidative stress is generally associated with all physiological anomalies in human. Several lines of evidence suggest that COVID-19 infection deteriorates in many patients with malnutrition, physiological stress and co-infections, all of which adversely alter the endogenous antioxidant machinery [5]. This cocktail factor adds to the evolving virulent mutants rapidly. Earlier studies have discussed the role of existing external factors in arising complication and thus mortality amongst the infected patients. These external factors not only include the existing environmental stress but also mental stress equally taking place in several patients due to the adverse situation of lockdown in COVID-19 [6]. The present SARS-CoV-2 and the previously known SARS-CoV viruses are evolved due to repeated mutations causing structural changes leading to varying virulence levels. Reported mutations in the spike (S) protein, i.e., D614G of SARS-CoV-2 multiply the rate of infection rendering existing medications ineffective [7]. Repeated SARS-CoV-2 mutations have further complicated the disease landscape (Figure 1). Similarly, point mutations in the receptor-binding domain (RBD) region have been reported in recent variant; remodeling the secondary structure and consequently infectivity [8]. In SARS-CoV-2 infection, virus mutation can lower down the immunity level in the affected patient [9]. Furthermore, increased levels of ROS in the body decreases T lymphocytes affecting the adaptive immune response.

Moreover, an inappropriate immune response characterized by excessive production of proinflammatory activators have been found, which results in common symptoms in severe COVID-19 patients. Increasing immunosuppression is another important source of endogenous oxidative stress resulting in imbalance in the oxidant-antioxidant ratio in the human body [10]. This situation elevates free radicals and thus enhances the reactive oxygen species (ROS). Elevated ROS in turn alters vital physiological process, triggers inflammation and promote further viral multiplication. This can be compensated by the use of different detoxifying enzymes, exogenous antioxidants from antioxidant rich dietary foods and nutrients. Polyphenols, a group of natural antioxidants are proposed to be used as potential adjuvants in therapy mainly due to their anti-inflammatory effect [11,12]. Iddir et al., clearly mentioned a direct correlation of the low nutrients with COVID-19 infection where deficiency in micronutrients like Zn and Vitamin A in diets increases the infection rate. Dietary constituents like polyphenols, vitamin C, vitamin E possess anti-inflammatory and antioxidant activity, which can neutralize the induced ROS and can interact with the inflammatory mediators in the infection [13].

Many known antiviral and antimalarial drugs like remdesivir and hydroxychloroquine are broadly studied for drug repositioning against COVID-19 infection. However, traditional medicinal plants also exhibit many prophylactic or preventive effects towards this disease. Needless to pinpoint the boon of these medicinal plants and their therapeutic effects. Since the time of Charaka Samhita in 1st millennium BC and inception of Ayurveda, the use of medicinal plants has been continuing for mitigation for various ailments with minimum side effects. Many ethnomedicinal use of the plants continue to be a choice of interest across the world [14,15]. The toxicity, pharmacological properties, and mode of actions of many medicinal plants have been studied in detail. The antioxidative property of noteworthy medicinal plants like *Curcuma longa*, *Nigella sativa*, *Oroxylum indicum*, *Echinacea purpurea*, *Cinchona* sp., *Glycyrrhiza glabra*, *Camellia sinensis* and various other medicinal plants have always proved to be potential candidates for major microbial infections [16,17,18]. Additionally, anti-inflammatory and antioxidative properties makes them a choice of interest for applications in treating COVID-19 [19,20]. Present review is an attempt to highlight the efficacies of some of these medicinal plants, preparations, and their important active constituents to be used in the arising complications in COVID-19.

## 2. Insight into the Pathogenesis of COVID-19

The inception of the novel coronavirus disease pandemic has engulfed more than 3,972,783 lives worldwide thus far [21]. There are now effective vaccines with a fairly high degree of success, however therapeutic drugs are still lacking. Also, before going into targeting this viral infection it’s an utmost urgency to better reveal the COVID-19 associated pathogenesis.

**Figure 2:**
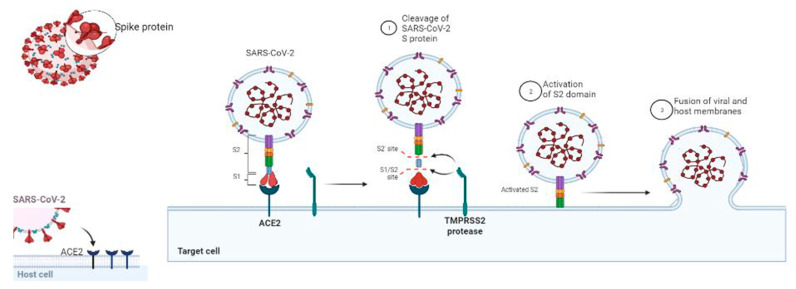
Pathogenesis of COVID-19 virus.

The viral architecture is a simple positive double stranded RNA makeup. The virus finds its entrance through the host angiotensin converting enzyme-2 (ACE-2). This transmembrane protein is abundant in the alveolar walls, lungs, epithelial cells and gastrointestinal tract. It makes complimentary binding site for the interaction with the viral spike protein [22,23]. This physiological process is triggered by S1 subunit of the spike protein by acidified proteolytic cleavage and assorted by transmembrane serine protease 2 (TMPRSS2) sufficient to complete the viral internalization process. This cleaves exposes various fusion peptides and helps in the formation of bundle across the cleavage site that results in union of the viral and host cell membrane; releasing viral genetic content into the host cytoplasm [24]. Once viral content is inside the cytoplasm, the virus gains access to process its infectivity and affect the lungs initially. The process of infectivity of COVID-19 virus is shown in Figure 2. Though little is known about the role of innate immunity, there is severe rush of cytokines shower, neutrophils, interleukin and C-reactive protein (CRP) release in the system with marked decrease in lymphocytes [25].

There is a high surge of IgM in the initial weeks of viral infection followed by increased IgG level in the latter phase during immunological response [26]. It multiplies within these cells then infects nearby cells, including alveolar macrophages [27,28]. Whole process of active response is triggered by apoptosis of the infected cells. This in turn activates proinflammatory cytokines, cell infiltration and tissue damage [29]. In COVID-19, increase of interleukins (IL-7, IL-2), inducible protein (IP)-10, granulocyte-colony stimulating factor, monocyte chemoattractant protein (MCP)-1 and tumor necrosis factor (TNF)-α are indicators of cytokine storm. Studies showed that a surge of cytokine storm in COVID-19 patients [29]. As entire scenario in COVID-19 patient is inter-dangled with inflammation, immunity and viral infection, anti-inflammatory activities and use of immune boosters can modulate the whole arising complexities.

## 3. Potential Targets of Medicinal Plants in COVID-19 Pathogenesis

Plant based natural products have always been a boon to cure many ailments since time immemorial. Their use as antiviral compounds is studied worldwide. Natural products in the form of nutraceuticals, medicinal foods, immune booster, antioxidants, and formulations showed antiviral activities. They have shown various modes of action including targeting viral attachment, its entry and multiplication, and suppressing inflammation [30]. Tangeretin, a citrus polymethoxyflavone compound, which is generally found in citrus peel oil is found to be greatly effective in COVID-19 treatment [31]. Similarly, other important plants like the *Curcumin longa*, *Oroxylum indicum*, *Echinacea purpurea*, *Cinchona* sp., *Glycyrrhiza glabra*, *Ocimum sanctum*, *Withania somnifera* and many others have reported immensely in treating COVID-19 disease. The bioactive compounds of medicinal plants can also alleviate the cytokines storm and inhibit the induced ROS, which subsequently can neutralize the inflammatory response. The role of medicinal plants and their potent molecules in different stages of COVID-19 pathogenesis intervention are summarized in Table 1.

### 3.1. Viral Attachment

All viruses of coronavirus group generally have similar pattern of spike protein and its route of entrance into the host. SARS-CoV-2 being a class of coronavirus family follows similar trend of entrance into the host cells. Earlier many reports of active compounds and its role in blocking the entrance have been narrated critically. This is either done by lowering the expression of the ACE-II receptors in the cells or by blocking the receptors by the natural compounds. *Glycyrrhiza glabra* is one such important medicinal plant found effective against SARS-CoV-2. Its active components like glycyrrhizin, and Glyasperin A are reported to have antiviral activity particularly against spike and non-structural protein-15 (nsp15). Molecular docking and simulation study showed higher binding efficacy of *G. glabra* extract that can inhibit the viral entry. Glyasperin A showed higher affinity towards spike glycoprotein [77]. Glycyrrhizic acid, another important compound of this medicinal plant has reported as protective agent in COVID-19 infection. Active compounds like Samarcadin, Femesiferol C, Famesiferol A, and Galbanic acid from *Ferula asafetida* have intensive binding affinities for ACE2 receptor [78]. Epigallocatechin gallate, common compound found in the green tea found potential against spike protein [79].

### 3.2. Encapsulation, Replication of the Virus and Viral Protease

In order to complete viral lifecycle, they involve the attachment to the active region of the host ACE-II receptor using the various virion surface glycoproteins. The entry mechanism of SARS CoV-2 depends primarily on TMPRSS2 and furin moieties, along with ACE-II receptor [80]. Therefore, inhibiting TMPRSS2 can be an effective treatment strategy. Another important protease is the chymotrypsin-like protease (3CLpro), which assists in the viral cleavage of already entered host cellular virus. It plays an important role in COVID-19 replication process [81]. Medicinal plant polyphenols, terpenoids and alkaloids can inhibit COVID-19 replication by targeting the activity of papain-like protease, 3CLpro, and fusion of the S protein of coronaviruses. Polyphenols inhibit signaling pathway responsible for these protease productions. Dietary polyphenols like chlorogenic acid, trigolnelline, caffeine, flavan-3-ols comprised in fractionated coffee, provide a natural source of antioxidants and interfere replication process of viral multiplications.

Additionally, the ability of the theaflavin, a component of black tea, in inhibiting the RNA dependent RNA polymerase (RdRP) active site is well documented in many recent works on COVID-19 research [82]. Bioactive compounds like vanicoside B, resveratrol, epicatechin, emodin, piceid, epicatechin from various medicinal plants including *Reynoutria japonica* and *Reynoutria sachalinensis* was found effective against the main protease (Mpro) [83]. Nimbin, gedunin, and ginsenosides, important constituents of *Panax ginseng*, *Zingiber officinale*, *Ocimum basilicum* are potential inhibitors of the main protease required for viral replication process of coronaviruses [84]. Punicalagin and punicalin, important compounds from *Punica granatum* peel extracts are reported to inhibit TMPRSS2, and ACE-II significantly [85]. *Andrographis paniculata* is reported to inhibit three potential key targets, papain-like receptors (PLpro), spike glycoprotein and the RdRp, which mediate the viral replication process. Neoandrographolide and 14-deoxy andrographolide, other active compounds of *A. paniculata* have shown to decrease 3CLpro and PLpro significantly [86].

Quercetin, the active flavanol common in many of the medicinal plants contains five hydroxyl moieties in its pharmacophoric region, which interferes and creates a hindrance in the early stages of the viral infections. It is usually achieved by blocking the angiotensin-converting enzyme quite significantly. The combinatorial activity of quercetin with zinc has also been reported where zinc has a role in the suppression of RdRp [87]. Some of the other important bioactive compounds having a direct role in inhibiting SARS-CoV-2 infection is briefly summarized in Table 1*. Nigella sativa* is another pharmacologically important plant, which contains a well-known bioactive principle thymoquinone. Being smaller in structure, this hydrophobic molecule aptly fits into the lipophilic envelope of SARS-CoV-2 inhibiting its entry and thereby replication [88].

### 3.3. Inflammation

COVID patients have surge of inflammation that is caused by increased macrophages, inflammasome and monocytes. Not only this, ROS also triggers the secondary inflammation that undergoes intercellular signaling. However, the exact mechanism is still being researched to understand how SARS-CoV-2 directly induces inflammation. It has been found that immune compromised patients have dysregulated NLRP3 inflammasome resulting in severe tissue damage and cytokine storm [89], indicating NLRP3 is one of the factors that mediates inflammation in these patients. This elevated inflammation further produces altered glycosylated IgG Fc tail and results in altered immune response. SARS-CoV-2 is independent to infect the monocytes resulting in the rush of production of proinflammatory cytokines in the mainstream to play its role in other secondary inflammation. Besides these various cytokines, chemokines, inflammatory transcription factors like NF-κB, and TNF-α play a pivotal part in inflammation associated with COVID-19 patients [90].

Traditional folklore varieties of medicinal plants are reported to have anti-inflammatory activities, which can alleviate these pro-inflammatory mediators. The anti-inflammatory activity of medicinal plants can check the cytokine storm in the affected patients and inhibit viral entry and replication [30]. Tangeretin, a citrus polymethoxyflavone compound, which is generally found in citrus peel oil is found to be effective in COVID-19 treatment [31]. *Cannabis sativa*, which has been used for various medical purposes, has shown anti-inflammatory activity associated with COVID-19 infection [91]. *C. sativa* extract fraction greatly reduced cytokines IL-6, and IL-8 as well as chemokines: C-C Motif Chemokine Ligands (CCLs)-2 and CCL7 levels, in an alveolar epithelial cell [91]. Limonene, a dietary terpene, has also shown to modulate inflammatory signaling pathways by inhibiting inflammatory mediators, like chemokines, cytokines and eicosanoids. Thus, limonene can act as inhibitor of inflammation in COVID-19 pathogenesis [92]. Flavonoids have a property to modulate cytokine levels, which indicates its possible role in COVID-19 associated inflammation. As it regulates inflammatory mediators, endothelial activation, and NLRP3 inflammasome, might be useful in regulating the cytokine storm [93].

Different formulations and preparation have also demonstrated anti-inflammatory activities in COVID-19 associated inflammation. The Ayurvedic formulation, Divya-Swasari-Vati, was found to suppress the pro-inflammatory cytokine levels, TNF-α and IL-6 both in humanized Zebrafish model [94]. ShufengJiedu capsules, an herbal drug with eight therapeutic plants, has also shown anti-inflammatory activity in the lung of HCoV-229E mouse model. It decreased inflammatory factors like the IL-10, TNF-α, IFN-γ and IL-6 in the lung [95]. Thus, COVID-19 mediated inflammation can be suppressed by traditional medicine formulations.

### 3.4. Oxidative Stress

Plants bestow antioxidant activities in a multifaceted manner, where their active ingredients work alone or in synergy to clear reactive oxygen species mainly by neutralizing them [10]. Many natural compounds were found to be efficient in earlier SARS related infections. Some of the phytocompounds were reported to inhibit the viral helicases rendering them ineffective. Several other notable plants including *Boerhavia procumbens*, *Cistanche tubulosa*, *Euphorbia hirta*, *Hyoscyamus niger*, *Capparis spinosa* L., *Carum copticum*, which not only decrease the inflammasomes in viral infection but also alleviate the level of ROS built due to cytokine storm [96]. Number of antioxidant molecules possess inhibitory function against inflammation, and viral infections. Astaxanthin, a known keto carotenoid also reported for its antioxidative efficacy in COVID-19 treatment. The polar moiety in the structure can leach the free radicals in and outside the cell membrane. This in turn enhances the process of attenuation of the stress mediated pathways in COVID-19 disease [97]. Similarly, the antiviral activities of two important antioxidant molecules, theaflavin and epigallocatechin-3-gallate (EGCG) are also reported to inhibit 3CLpro [98,99]. This explains the antioxidant medicinal plants not only lower the internal ROS but can directly interfere with the viral replications by modifying different helicases and proteases.

### 3.5. Immune Response

Immunity gets compromised in the later stages of this novel coronavirus disease. The decreased immune response causes increased secondary complications and resulting in organ failure in the suffering patients. Medicinal plants have always been used as a combinatorial medicine with other phytochemicals. Their antioxidative efficacy boosts up the immunity level in the patients to minimize further complexities in the COVID-19 disease. Ginseng plant has immunostimulating properties. They can induce cytokine productions activating the macrophages and enhancing the antibody productions [100]. Achyranthes root popularly called the Chinese yam also reported to increase the antibody productions [101]. Many recent researches on tea polyphenol showed its efficacy to modulate and augment innate immunity to prevent COVID-19 [102]. Plant based rich fiber diet are found to be beneficial, as it enriches the intestinal and gut microbiome of the host which enhances immunity significantly. This enhanced immunity has shown to be resulted in better prognosis of patients with COVID-19 [103].

Compounds derived from *Allium sativum* (garlic) have been reported to alleviate cytokine level and reverse the immunological abnormalities to comparatively safer situation in COVID-19 patients. Thus, *A. sativum* can be an important plant against SARS-CoV-2 infection [104]. Besides these, several other medicinal plants including *Echinacea* spp., *Boswellia* spp., *Glycyrrhiza* spp., *Curcuma longa,* medicinal fungi, *Pelargonium sidoides* and *Sambucus* spp. are documented with immunomodulatory, immunostimulatory, and anti-inflammatory effects. These immune responsive properties of medicinal plants can help to prevent the viral infections [105]. Thus, these natural immune boosters can help improve various complications associated with the COVID-19 disease.

## 4. Effect of Medicinal Plants in COVID-19 Associated Secondary Diseases

**Figure 3:**
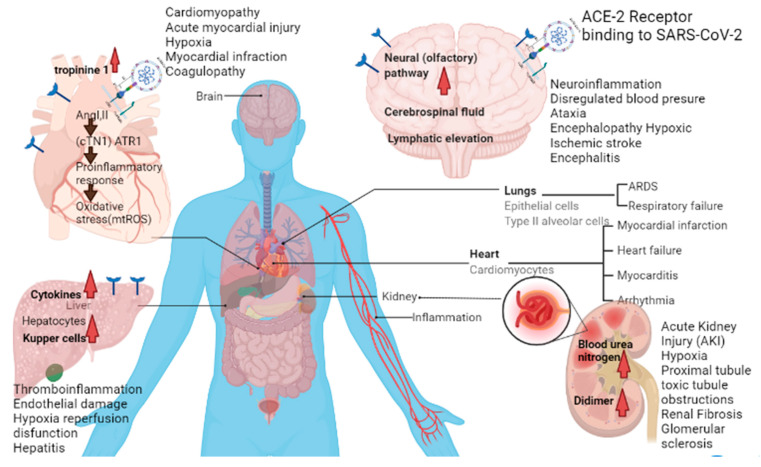
Post-COVID-19 conditions that may occur in different organs.

It is seen in many cases that COVID-19 patients experiences post-COVID complications. However, most of the patients recover in 1–2 weeks and have fairly any emerging complications. Post-COVID conditions are wide range of complications of different organs including but not limited to lungs, liver, kidney, heart, brain (Figure 3) that patients can experience several weeks after infection. Some of the post-COVID conditions with their phytotherapy are described below (Table 2).

### 4.1. Acute Kidney Damage

Renal symptoms including acute kidney injury (AKI) is one of the most frequently occurring COVID-19 associated secondary complications [143]. In a study with 3993 hospitalized COVID-19 patients, AKI has shown to occur in 1835 (46%) [144]. The pathways of SARS-CoV-2 mediated kidney damage is still in process of understanding but some clinical and experimental data indicated that it may be caused by viral binding with ACE2 receptor of kidney cells considering that normal kidney cells exhibited higher expression of ACE2 receptor than lung cells [145]. After binding to ACE2 receptor the COVID-19/ACE2 complex generally takes entry to the host cell by endocytosis, those which are unable to take entry to host cell shed by ADAM Metallopeptidase Domain 17 (ADAM17) [146]. Moreover, elevated level of ADAM17 in renal tissue is thought to orchestrate angiotensin II-induced renal lesion in the chronic kidney disease patients [147].

Interestingly, kidney biopsies of Afro-American COVID-19 patients appeared to be collapsing focal segmental glomerulosclerosis with elevated level of APOL1, resulting in functional effect of podocytes due the viral replication [148,149]. However, the undeviating SARS-CoV-2 entry through CD147, another transmembrane receptor, instigates cyclophilin mediated renal inflammation [150]. Li et al. in a comprehensive clinical study assessed the kidney function of 193 COVID patients among which 31% of patients had increased blood urea nitrogen (BUN) level, 22% with high creatinine level and 70% with elevated level of D-dimer. The incidence of proteinuria and hematuria was also seen in many COVID-19 patients [151]. Additionally Su et al. when analyzing the renal tissue of COVID-19 infected patients found increased damage rates in proximal tubules with marked erythrocyte aggregation and vacuolization in the glomerular and peritubular capillaries [150]. There is a strong chance that post SARS-CoV-2 infection may give rise to cytokine storm including IL-6, CRP and TGF-ß can contribute to lung-kidney crosstalk leading to AKI [152]. A current study has directed that SARS-CoV-2 triggered immune reaction is instructed by TGF- ß [153].

Natural compounds have shown to be very helpful in the management of AKI induced by COVID-19. Quercetin, which is found in wide range of medicinal plants and dietary supplements, has found to be safe and potential candidate against AKI induced by different nephrotoxic agents. Using network pharmacology and molecular docking, it has been observed that quercetin exhibits renal protective effects on COVID-19-induced AKI through inhibiting the signaling pathways associated with apoptosis and inflammation. Quercetin also inhibits SARS-CoV-2 main protease 3CL and ACE2, suppressing the integral proteins and thus affects the viral life cycle [154]. As increased level of oxidative stress and inflammation are found in COVID-19 patients, quercetin has found to be nephroprotective by suppressing ROS and inflammation induced AKI [155].

Lingustrazine (tetramethylpyrazine), a natural product isolated from Chinese herb, significantly diminished the mRNA levels of TGF-β1 and also inhibited the loss of cytokeratin-18 expression hence attenuate renal tubulointerstitial fibrosis [112]. Another important but less explored species *Salvia multiorrhiza* (family Lamiaceae) commonly known as Danshen is rich in tanshinones and salvionalic acids. Tanshinone IIA is well known for modulating TGF-𝛽 signaling pathway and anti-inflammatory effect. Using AKI model, Jiang and coworkers showed that Tanshinone IIA markedly reduces the expression of TGF-β1 and MCP-1 [109]. Similarly, Salvianolic acid A are found to inhibit inflammatory cytokines and Smad signaling pathway and thus prove to be effective in renal fibrosis [111]. As TGF-β and cytokines are highly expressed in kidney cells of COVID-19 patients, suppression of these molecules can help in the improvement of COVID-19 patients.

### 4.2. Cardiovascular Disease

While the clinical exemplifications of COVID-19 disease are generally subjugated by respiratory symptoms, 8–12% of total patients were diagnosed with increased levels of cardiac troponine [156]. A recent clinical case study with 21 COVID-19 patients has disclosed that 42.9% of them were found to encounter congestive heart failure [157]. Another clinical study with 187 COVID-19 patients showed that 35% of them had comorbidities like hypertension, cardiomyopathy and 28% exhibited acute myocardial injury which was characterized by high levels of cardiac troponin I (cTnI) [158]. However, there is lack of exact etiological evidence that could highlight the detailed mechanism of SARS-CoV-2 and cardiac tissue interaction. Considering that ACE2 receptor is abundantly present in myocytes may indicate the direct entry of SARS-CoV-2 into heart. The ACE2 receptor plays a critical role in neurohumoral regulation of cardiovascular system. The direct binding of SARS-CoV-2 with the cardiac ACE2 receptor seems to result in acute myocardiac injury. Upon viral entry the systemic inflammation coupled with hypoxia can alter myocardial oxygen demand-supply ratio and can also induce plaque rupture and occurrence of myocardial infarction [159].

As overexpression of ACE2 receptor along with angiotensin in heart muscle cells causes systemic inflammation and myocardiac injury in COVID-19 patients, targeting these molecules by natural compounds can relive the infection associated cardiovascular illness. Studies conducted on rat model showed that *Ulmus wallichiana* plant extract has ability to decrease renin, Ang II, ACE activity as well as level of inflammatory molecules, which indicates its cardioprotective effect [160]. Another phytochemical rosmarinic acid, a major active constituent of *Rosmarinus officinalis*, exhibited cardioprotection on myocardial infarction-induced cardiac fibrosis in rat model through the suppression of ACE and angiotensin type 1 receptor (AT1R) expression [160]. Ang II, ACE and AT1 receptor are also found to be suppressed by ginsenoside Rb1; major component of the genus Panux plant in rat model. This suppression by ginsenoside Rb1 led to the improvement in cardiac function as indicated by attenuation in cardiac hypertrophy and myocardial fibrosis [120]. Besides angiotensin II levels, natural compounds also exert cardioprotective by suppressing inflammatory markers. In a study, blueberry extract decreased interleukin 6, endothelin 1, malondialdehyde, and angiotensin II levels and prevented particular matter (PM2.5)-induced cardiovascular damage in rats [161]. In another study, orange beverage has shown to increase the antioxidant levels and maintain the levels of inflammatory markers IL-6 and C-reactive protein in animal model, and thus exhibited protection against cardiovascular risk factors [162]. These studies demonstrate that phytochemicals have the ability to reduce COVID-19 infection-induced enzymes, receptors and inflammatory markers level in cardiovascular system and suppress the occurrence of myocardial injury in the patients.

### 4.3. Respiratory Disease

The notable clinical manifestation of COVID-19 includes pneumonia, severe lung inflammation and acute respiratory distress syndrome (ARDS) leading to breathing problem in affected patients. Apart from this, the severity may outstretch to a complete shutdown of lung functions and induction of sepsis. Although a large proportion of clinical and experimental research are focusing on repurposing and combinatorial use of FDA approved drugs, efforts have been also made for the search of plant derived anti-viral compounds. SARS-CoV-2 enters into human host via the ACE2 receptor, which is highly expressed in lung tissue. Besides this, inflammation of the lungs is also the main factor leading to respiratory distress in patients with severe COVID-19.

In view of this, attempts have been made to identify natural inhibitors of inflammation, ACE2 and 3CL protease. However, extract of *Andrographis paniculata* and its active compound andrographolide directly inhibit the infectivity of SARS-CoV-2 in lung epithelial Calu-3 cells with an IC_50_ of 0.036 μg/mL and 0.034 μM, respectively [163]. Further, using artificial 3D human models of various tissues including lung airway, it has been found that *Cannabis sativa* extracts decreases ACE2 expression levels [164]. *Prunus mahaleb* L. fruit also downregulates the gene expression of ACE2 and TMPRSS2 in lung epithelial H1299 cells, suggests the potential role in inhibiting SARS-CoV-2 entry in the human host [165]. Besides ACE2 protein, *C. sativa* has also shown to reduce IL-6, IL-8, CCL2 and CCL7, expression in the lung epithelial A549 cell line and thus anticipated to prevent or treat COVID-19 infection [91].

A formulation tri-herbs, which contains ursolic acid, withanone, withaferine, cordifolioside A, magnoflorine, withanoside IV-V, betulinic acid, rosmarinic acid, and palmatine as phyto-metabolites, attenuated the inflammatory markers such as TNF-α and IL-6, and decreased the NF-κB/AP-1 transcriptional activity in lung cells, suggesting its potential use in SARS-CoV-2 infection [94]. ShufengJiedu formulation, which comprised of multiple medicinal plants, has shown to decrease virus load and increase immune response in the lungs of mouse model, in addition to decreasing inflammatory factors [95]. The (-)-epigallocatechin-3-gallate (EGCG), a major constituent of green tea, has also shown potential to inhibit SARS-CoV-2 life cycle. It inhibits cytokine storm-mediated lung injury, acute respiratory distress syndrome, and lung fibrosis through increasing Nrf2 and suppressing NF-κB inflammatory signalling [98].

### 4.4. Neurological Disease

The pre-Bötzinger complex (PBC) in the brain primarily functions as respiratory oscillator. It might be possible that during the infection, SARS-CoV-2 targets central nervous system and inhibits the PBC in the brainstem thus contributing respiratory breakdown [166]. In COVID-19 outbreak, the accumulating evidence of clinical research stipulate that COVID-19 affected patients develop several neurological complications. Out of which are directly associated with SARS-CoV-2 infection [167]. Several clinical analysis and data obtained from patients medical record established the prevalence of headache, altered mental status, disturbance in consciousness with acute cerebrovascular disease [168,169,170]. A study analyzed in 219 COVID-19 patients where 10 of them (4.6%) were found to develop acute ischemic stroke and 1 patient (0.5%) encountered with intracerebral hemorrhage [171]. Analysis of 76 patients conducted by Scullen and coworkers where 20 patients (74%) were diagnosed with COVID-19 associated encephalopathy and 2 (7%) with COVID-19–associated acute necrotizing encephalopathy [172]. Apart from these a very few cases of rare neurological diseases Guillain–Barré syndrome and Miller Fisher Syndrome were also encountered [173,174]. A recent comprehensive study was published in Lancet Psychiatry reported an average of 33.33% COVID-19 patients are diagnosed with a neuropsychiatric condition in the next six months which include dementia, anxiety and depression [175].

It has been revealed that many of the traditionally used medicinal plants have the property to overcome COVID-19 associated neurological disorders. In this light, Ayurvedic plants not only boost the immunity but also directly affect the symptoms of depression or anxiety in COVID-19 patients [176]. Luteolin, present in several plants, has shown to suppress neuroinflammatory responses in COVID-19 and exhibited neuroprotective effects through various mechanisms through inhibition of various immune modulatory cells [177]. Luteolin formulation together with the antihistamine rupatadine also shown to inhibit brain fog and other neuropsychiatric symptoms that have been implicated in the pathogenesis of cytokine storms in COVID-19 patients [178].

### 4.5. Liver Disease

Generally the chronic liver disease (CLD) is correlated with immune dysregulation and inflammation, which may present a greater risk of COVID-19 mediated pathogenesis [179]. Recent shreds of evidence support the cross talk between SARS-CoV-2 infection and CLD. Some clinical studies have divulged that patient with COVID-19 symptoms are having elevated level of aspartate transferase (AST), alanine transferase (ALT), alkaline phosphatase (ALP) and gamma-glutamyltransferase (GGT). High level of these markers is generally correlated with liver injury [179,180,181]. A recent clinical report revealed that 2–11% of overall COVID-19 patients have underlying CLD and 14–53% with COVID-19 associated hepatic dysfunction [182]. An experimental outcome by Zhao et al. suggested that SARS-CoV-2 infected human liver ductal organoids showed elevated level of viral mRNA. They further performed transcriptome analysis and found that about 51.45% of total cells expressed TMPRSS2 receptor [183]. Also, membrane less SARS-CoV-2 vesicles have been recognized in the hepatocyte’s cytoplasm of two COVID-19 patients along with high expression of liver damage markers [184]. The SARS-CoV-2 mediated hypoxia and cytokine storm could also potentiate liver injury [179]. Meanwhile some other studies reported that antiviral drugs used for the support of COVID-19 patients could also induce hepatotoxicity. Drugs like remdesivir, hydroxychloroquine and acetaminophen which are being commonly used in COVID-19 patients may be responsible for liver toxicity [185,186,187].

Therefore, natural products could have therapeutic potential in the management of COVID-19 affected liver injury. Based on the available evidence, glycyrrhizin preparations have been shown to be a useful therapeutic agent for SARS-CoV-2 infections, especially those complicated with liver damage [188]. In a study with 147 COVID-19 patients, it was found around 38.1% had anomalous ALT level and 54.4% had unusual AST expression. Patients had also increased serum LDH levels and decreased SOD levels. However, treatment with diammonium glycyrrhizinate preparation alleviated the abnormal liver enzyme activities in non-critical COVID-19 patients [189]. It’s another preparation Diammonium glycyrrhizinate has shown to reduce acute liver injury in patients with COVID-19 [190]. Further, a study on EGYVIR (combined formulation of black pepper and curcumin extracts) has shown that it inhibits COVID-19 infection-associated inflammatory NF-κB factor and cytokine storm in hepatic Huh-7 cell line [191]. Thus, natural compounds have the potential to hinder the production of inflammatory factors and improve the liver enzyme activities. Therefore, the effect of these pharmaceutically important plants and their active isolates could be also explored for the management of COVID-19 associated liver disease.

### 4.6. Others

Besides aforementioned secondary and post COVID-19 disease, many patients continue to experience symptoms such as anxiety, depression, fatigue, insomnia and also muscular sprain. However, facts from pre-pandemic observational and other related studies suggested that a nutraceutical may provide benefit to the patients with COVID-19 in managing these disorders [192] Ayurvedic medicines have also found to beneficial in curing COVID-19 associated symptoms [193]. Korean medicine was also found to be effective in managing COVID-19 associated symptoms. In a clinical trial with COVID-19 patients, treatment of Kyung-Ok-Ko (*n* =2285) herbal medicine, and QingfeiPaidu decoction (*n* = 2053) led to improve on COVID-19-related symptoms like dry cough, headache, muscle pain, sore throat, dyspnea and loss of appetite. As corticosteroids have been administrated to severe patients with COVID-19, resulted in osteonecrosis of the femoral head (ONFH) disabling complication among convalescent SARS patients. However, Chinese herbal Huo-Gu formula therapy demonstrated beneficial effects on preventing femoral head collapse, delaying total hip arthroplasty, and maintaining physical function caused by ONFH in COVID-19 patients [194]. Besides these, several traditional medicines and herbal preparations have been used as therapeutic option for treating COVID-19 associated symptoms and post recovery.

## 5. Conclusions

Hardly any country remains untouched by the menace of COVID-19 pandemic and its deleterious outcome. In this situation of crisis, it is utmost necessary to control the present ongoing condition. Where bringing out newer drugs is relatively harder it is very urgent to bring about a quick remedy for this disease. Medicinal plants have always been a huge repository presenting vivid range of natural products having both prophylactic and preventive activity against many serious complications. Where pooling the entire bioactivity data is a tedious job amidst lots of existing extracts variation and lack of proper standardization of the purified extracts, so direct anti-inflammatory, antioxidant and antiviral activity of such medicinal plants can be directed to understand their protective role in the mitigation of the various prevailing secondary complications associated with the COVID-19. Important plants like *Withania somnifera*, *Ginkgo biloba*, *Citrus reticulata*, *Camellia sinensis*, *Glycyrrhiza glabra*, *Coffea arabica* mentioned here in this review are rich in antioxidant and inflammatory activity. They show inhibitory action against integral proteins of SARS-CoV-2, whether it’s the spike protein or the inhibition of the RNA dependent polymerase, or papain like receptor and ACE-II receptor. So, further in-depth study on these medicinal plants with antioxidant and inflammatory activities can be studied for more putative compounds that can be promising leads in the development of newer therapeutic drugs.

## Figures and Tables

**Table 1:** Targeting COVID-19 associated molecules by medicinal plants and their bioactivities.

Medicinal Plants	Plant Active Compound	Antiviral Activity	References
*Citrus aurantium*	Hesperidin	Inhibition of main protease (M^pro^)	[32]
*Camellia sinensis*	Epigallocatechin gallate	Inhibition of spike glycoprotein (S-protein)	[33]
*Linaria vulgaris*	Pectolinarin	Inhibition of spike glycoprotein and M^pro^	[33]
*Rhus succedanea*	Rhoifolin	Inhibition of M^pro^	[33]
*Coffea arabica*	Epigallocatechin gallate	Inhibition of M^pro^	[33]
*Cirsium japonicum*	Cirsimaritin	Inhibits against M^pro^	[34]
*Glycyrrhiza glabra*	Glycyrrhizin	Reduces the level of TMPRSS2	[35]
*Acalypha australis*	Emodin	ACE-II Inhibitors	[36]
*Citrus reticulata*	Nobeletin	ACE-II Inhibitors	[36]
*Lycoris squamigera*	Lycorine	Inhibition of RdRP activity	[37]
*Stephania cepharantha*	Cepharanthine	Inhibition of viral-target binding	[38]
*Citrus bergamia*	Naringenin	ACE-II Inhibitors	[36]
*Justicia procumbens*	Justicidin D	Inhibition of RdRP and spike protein	[39]
*Oroxylum indicum*	Baicalin	ACE-II Inhibitors	[36]
*Curcuma longa*	Curcumin	Inhibits against M^pro^activity	[34]
*Morus alba*	Moralbanone	Inhibition of SARS-3CL^pro^	[40]
*Linum usitatissimum*	Herbacetin	Inhibits the activity of 3CL^pro^	[41]
*Maclura tinctoria*	Morin	Inhibition of M^pro^ and S-protein	[33]
*Cirsium chanroenicum*	Pectolinarin	Inhibition of M^pro^ and S-protein	[33]
*Scutellaria baicalensis*	Scutellarin	Inhibition of SARS-3CL^pro^	[42]
*Taraxacum officinale*	Epitaraxerol	Inhibits viral activity	[43]
*Ginkgo biloba*	Kaempferol	Inhibition of SARS-3CL^pro^ activity	[44]
*Berberis buxifolia*	Berberine	Targets SARS-Cov-2 M^pro^ activity	[45]
*Phyllostachys nigra*	Vitexine	Targets SARS-Cov-2 M^pro^activity	[45]
*Toddalia asiatica (Linn.) Lam.*	Toddacoumaquinone	Targets SARS-Cov-2 M^pro^activity	[46]
*Olea europaea*	Oleuropein	RdRP inhibitors	[47]
*Vitis vinifera*	Resveratrol	RdRP inhibitors	[47]
*Caryophyllales* sp.	Bisbenzylisoquinoline	Suppressing viral entry	[48]
*Oroxylum indicum*	Baicelin	Inhibits the activity of 3CL^pro^	[49]
*Tinospora cordifolia*	Cordifolide A	Inhibition of M^pro^ and S-protein	[50]
*Withania somnifera*	Withanone	Inhibits the active protease M^pro^	[51]
*Withania somnifera*	Withanolide B	Inhibits papain like receptor and ACE-II receptor	[52]
*Origanum vulgare*	Carvacrol	Inhibits main protease (M^pro^) and SARS-CoV-2 entry	[53]
*Caesalpinia minax.*	Bonducellpin D	Inhibition of M^pro^	[54]
*Salvia officinalis* L.	Rosemarinic acid	Inhibits the activity of 3CL^pro^	[55]
*Thymus vulgaris* L.	Thymol	Inhibits spike glycoprotein	[56]
*Nepeta cataria*	Pulegone	Inhibits spike glycoprotein	[56]
*Glycyrrhiza glabra*	Glabiridin	Inhibits the main protease	[57]
*Fagopyrum esculentum*	Rutin	Inhibition of the RNA dependent polymerase	[58]
*Cymbopogon winterianus*	Citronellol	ACE-II Inhibitors	[59]
*Torreya nucifera* L.	Bilobetin, amentoflavonone	Inhibition of M^pro^	[60]
*Lindera aggregate*	Linderane and linderalactone	Inhibition of 3CL^pro^ and spike protein	[61]
*Asparagus racemosus*	Asparoside-C, Asparoside-D and Asparoside -F	Inhibition of spike protein and nsp 15	[62]
*Citrus reticulata*	Hesperitin, hesperidin	Inhibition of 3CL^pro^	[63]
*Isatis indigotica*	Hesperitin, hesperidin, rutin, phaitanthrin D	Inhibition of 3CL^pro^	[64,65]
*Cinnamomum zeylanicum*	Apigenin, geranylated flavonoids	Reduction in transcription factor NF-κB activation	[66]
*Panax ginseng*	Polysaccharides	Immunomodulatory properties	[67,68]
*Urtica dioica*	*β*-sitosterol, tannic acid	Inhibitor of ACE-II receptor	[69]
*Houttuynia cordata*	Bavachinin, cosmosilin,	SARS-3CLproinhibition, RdRP	[70]
*Cibotium barometz*	Neohesperidin	Inhibition SARS-3CL protease	[71]
*Hibiscus sabdariffa* L.	Anthocyanin. cyanidine, anastatin A	Inhibition SARS-3CL protease	[72]
*Salvia officinalis*	Luteolin, caffeic acid,	Inhibits viral replication	[73]
*Allium sativum*	Alliin, diayl disulfide, allicin, quercetin	Decreasing of viral infection rate and interacts with M^pro^	[74]
*Mentha piperita*	Terpenoids and menthol	Inhibition of acute respiratory infection	[75]
*Morus alba*	Moralbanone, mulberroside C, eudraflavone B	Inhibition of viral replication	[40]
*Justicia adhatoda*	Anisotine, vasicoline, pemirolast	Inhibition of RdRP	[76]

**Table 2:** Post-COVID conditions with their phytotherapy.

Natural Compounds	Source	Pathology	Mechanism	Effective Concentration	References
**Kidney damage**					
Poricoic acid ZA	*Poriacocos*	Renal fibrosis (RF)	Inhibits Smad2/3 phosphorylation	10 µM	[106]
poricoic acid ZG	*Poriacocos*	RF	Inhibits Smad3 and alter TGF-β/Smad signaling	10 µM	[107]
poricoic acid ZH	*Poriacocos*	RF	Inhibits Wnt or β-catenin signaling	10 µM	[107]
25-O-methylalisol	*Alisma rhizoma*	RF	Inhibits Wnt or β-catenin signaling	10 µM	[108]
Tanshinone IIA	*Salvia multiorrhiza*	Acute kidney injury	Inhibits TGF-β1 and MCP-1 expression	15 mg/kg	[109]
Leonurine	*Leonurus* spp.	RF	Inhibits TGF-β/Smad pathway	50 mg/kg/day	[110]
Salvianolic acid A	*Salvia multiorrhiza*	RF	Inhibits TGF-β1/Smad signaling and inflammatory cytokines	17.1 mg/kg	[111]
Ligustrazine	*Ligusticum striatum*	RF	Inhibits TGF-β pathway	80 mg/kg	[112]
Saikosaponin-D	*Bupleurum falcatum*	Oxidative stress	Increases antioxidant proteins CAT, SOD1, HSP72 and GPx-1	3 µg/mL	[113]
Triptolide	*Tripterygium wilfordii*	RF	Decreases TGF-β and MCP-1 expression	0.6 mg/kg	[114]
Sulforaphane	*Brassica oleracea*	Renal ischemia	Increases the level of Nrf2 and NQO-1	500 µg/kg	[115]
**Cardiovascular disease**					
Malvidin-3-glucoside	*Vitis* spp.	Endothelial dysfunction	Reduces iNOS, COX-2, IL-6 through inhibition of NF-kβ activation	25 µM	[116]
Sinigrin	*Brassica* spp.	Atheresclerosis	Attenuates VCAM-1, ICAM-1, CCL2, CCL5 expression and reduces LDH, LDL concentration in serum	10 mg/kg	[117]
Delphinidin	*Delphinium* spp.	Heart ischemia	Rapid reduction of cytochrome c	40 µM	[118]
Cyanidin	*Vaccinium* spp.	Heart ischemia	Rapid reduction of cytochrome c	40 µM	[118]
Thymoquinone	*Nigella sativa*	Cardiac failure	Decreases oxidative and nitrosative stress	200 mg/kg	[119]
Ginsenoside Rb1	*Panax* spp.	Cardiac failure	Reduces the level of ANF, β-MHC, Ang II, ACE, AT1 and enhances translocation of GLUT4 to plasma membrane	35 mg/kg, 70 mg/kg	[120]
Phlorotannin	*Fucus spiralis*	High blood pressure.	Inhibits ACE	200 µg/mL	[121]
Oleocanthal	*Olea europaea*	Atherosclerosis	Inhibition of platelet aggregation mediated inflammation	40 mL of extra virgin olive oil	[122]
Barberine	*Berberis* spp.	Myocardial ischemia	Decreases CK-MB, LDH, TNF-α, IL-6 and regulate HMGB1-TLR4 axis	30 mg/kg, 60 mg/kg	[123]
Resveratrol	*Vitis* spp.	Myocardial ischemia	Promotes VEGF-B/ antioxidant signaling pathway	10 µM	[124]
**Liver injury**					
Glycyrrhizin	*Glycyrrhiza glabra*	Hepatic ischemia-reperfusion	Inhibits Gasdermin D-mediated pyroptotic cell death of Kupffer cells	100 uM	[125]
Chicoric acid	*Cichorium intybus*	Hepatitis B	Block viral protein and DNA replication	100 µg/mL	[126]
Curcumin	*Curcuma longa*	Liver cirrhosis	Reduces oxidative stress	300 mg/kg/day	[127]
Dieckol	*Ecklonia cava*	Carbon tetrachloride induced liver damage	Upregulates antioxidant enzymes	25 mg/kg	[128]
Puerarin	*Pueraria lobata*	Carbon tetrachloride induced liver damage	Regulates JNK/c-Jun/CYP7A1 Pathway	400 mg/kg	[129]
Delphinidin	*Delphinium*	Hepatitis C	Impairs viral attachment to cell	100 µM	[130]
Gallic acid	*Fragaria ananassa*	Paracetamol induced liver damage	Decreases TNF-α and lipid peroxidation levels	100 mg/kg	[131]
Baicalein	*Scutellaria* *baicalensis*	Carbon tetrachloride induced liver damage	Decreases AST and ALT levels	80 mg/kg	[132]
Troxerutin	*Styphnolobium* *japonicum*	Liver inflammation	Reduces oxidative stress mediated NAD+-depletion	150 mg/kg/day	[133]
**Neurological disorder**					
Resveratrol	*Vitis* spp.	Cerebral ishchemia	Enhances Nrf2 and HO-1 expression	30 mg/kg	[134]
Kaempferol	*Brassica oleracea*	Oxidative stress induced neurotoxicity	Inhibits GSK3β and enhance Nrf2 expression	21 mg/kg	[135]
Ginkgolide	*Ginkgo biloba*	Cerebral ishchemia-repurfusion injury	Elevates the TNF related weak initiator of apoptosis (TWEAK) ligand	2.5 mL/kg	[136]
Pterostilbene	*Pterocarpus marsupium*	Oxidative stress induced neurotoxicity	Acts as estrogen like compound to activate estrogen receptor-α (ER-α) mediated signaling	10 nM	[137]
Myricanol	*Myrica rubra*	Oxidative stress induced neurotoxicity	Enhances Nrf2 expression	50 mg/mL	[138]
Capsaicin	*Capsicum annuum*	Cerebral ischemia	Regulates transient receptor potential channel vanilloid subfamily member 1 (VR1)	0.2 mg/kg	[139]
Ergothioneine	*Pleurotus ostreatus*	Endothelial Injury	Decreases IL-8, IL-6, TNF-α, COX2 expression	10 µM	[140]
Epigallocatechin-3-gallate	*Camellia sinensis*	Neuroinflammation	Alleviates the STAT3 and IL-6 levels	50 mg/mL	[141]
Cannabigerol& cannabidiol	*Cannabis sativa*	Neuroinflammation	Reduces NF-kB activation and increase Nrf2 levels.	5 µM	[142]

## Data Availability

Not applicable.
